# Risk factors and a new nomogram for predicting brain metastasis from lung cancer: a retrospective study

**DOI:** 10.3389/fonc.2023.1092721

**Published:** 2023-06-19

**Authors:** Bo Wu, Yujun Zhou, Yong Yang, Dong Zhou

**Affiliations:** ^1^ The Second School of Clinical Medicine, Southern Medical University, Guangzhou, China; ^2^ Department of Neurosurgery, Guangdong Provincial People's Hospital (Guangdong Academy of Medical Sciences), Southern Medical University, Guangzhou, Guangdong, China

**Keywords:** lung cancer, brain metastasis, risk factors, nomogram, gender, pathological type, leukocyte count, fibrinogen stage

## Abstract

**Objective:**

This study aims to establish and validate a new nomogram for predicting brain metastasis from lung cancer by integrating data.

**Methods:**

266 patients diagnosed as lung cancer between 2016 and 2018 were collected from Guangdong Academy of Medical Sciences. The first 70% of patients were designated as the primary cohort and the remaining patients were identified as the internal validation cohort. Univariate and multivariable logistics regression were applied to analyze the risk factors. Independent risk factors were used to construct nomogram. C-index was used to evaluate the prediction effect of nomogram.100 patients diagnosed as lung cancer between 2018 and 2019 were collected for external validation cohorts. The evaluation of nomogram was carried out through the distinction and calibration in the internal validation cohort and external validation cohort.

**Results:**

166 patients were diagnosed with brain metastasis among the 266 patients. The gender, pathological type (PAT), leukocyte count (LCC) and Fibrinogen stage (FibS) were independent risk factors of brain metastasis. A novel nomogram has been developed in this study showed an effective discriminative ability to predict the probability of lung cancer patients with brain metastasis, the C-index was 0.811.

**Conclusion:**

Our research provides a novel model that can be used for predicting brain metastasis of lung cancer patients, thus providing more credible evidence for clinical decision-making.

## Introduction

With the supreme morbidity and mortality, lung cancer is still the most malignant growth at present. The cumulative incidence of brain metastasis (BMs) in lung cancer in 1 year and 5 years is 14.8% and 16.3% (1), respectively, which is the first incidence of all types of tumors. In addition, 80-85% of lung cancer patients are diagnosed as non-small cell lung cancer(NSCLC) (2), so NSCLC is the most familiar primary neoplasm in brain metastasis. We found in some research that the rate of brain metastasis with NSCLC is about 10% to 20% (3–5), and the rate is even higher in advanced NSCLC, about 30% to 50% (4, 6–8). The median survival of brain metastasis is about 6 to 30 months, but early diagnosis is propitious to prolong the survival time (3, 4). Therefore, it’s positive for patients if clinicians can determine the probability of brain metastasis early.

At present, a great many essays have been issued to analyze the risk factors for brain metastasis of lung cancer (9, 10). However, their assessment criteria are more complex. In this study, we aimed to analyze the risk factors for brain metastasis in lung cancer patients and establish an effective and noninvasive nomogram for the possibility of brain metastasis in lung cancer patients by adopting advanced statistical analysis. In our nomogram, we can speculate the possibility of brain metastasis through simple blood routine and pathological type, and this nomogram is easier to apply to clinical practice than others of the same type.

## Materials and methods

Clinical data of all patients was uninterruptedly enrolled and this study was ratified by the Guangdong Provincial People’s Hospital. All enrolled patients were carefully screened according to the following inclusion criteria: Group of primary lung cancer:(a) Patients diagnosed with pathological results;(b) Patients diagnosed only primary neoplasms without brain metastasis;(c) Patients diagnosed between 2016 and 2018;(d) Patients with completely clinical characteristics. Group of brain metastasis:(a) Patients diagnosed with pathological results;(b) Patients diagnosed between 2016 and 2018;(c) Patients with completely clinical characteristics. Finally, a total of 100 patients with primary lung cancer and 166 patients with brain metastasis who were diagnosed at the Department of Neurosurgery, the Guangdong Provincial People’s Hospital from 2016 to 2018 were enrolled in this retrospective study.

All patients were randomly arranged. The first 70% of patients were designated as the primary cohort, and the remaining patients were identified as the internal validation cohort. The verification of the nomogram was also assessment in an independent external validation cohort which included 100 patients from 2018 to 2019 who was diagnosed with primary lung cancer and brain metastasis.

### Clinical characteristics

As shown in **Table 1**, the clinical characteristics include gender, age, pathological type (PAT), location of primary tumor, smoke, drunk, blood routine tests, glucose count, protein count, albumin count, electrolyte and coagulation indicators were obtained from medical records. The blood routine tests include erythrocyte count, leukocyte count (LCC), platelet, eosinophil ratio count, lymphocyte ratio count, monocyte ratio count and neutrophil ratio count, basophil ratio count. The electrolyte including kalium count, natrium count, chlorine count, calcium count, magnesium count, phosphorus count. And the coagulation indicators including Activates partial thromboplastin time (APTT), Fibrinogen count (FibC), Thrombin time (TT), International normalized ratio (INR), Prothrombin activity (PTA) and Plasma prothrombin time determination (PT). This study divides all the continuous variables into low, medium, and high groups with the third point as the dividing line.

**Table 1:** Detail of patients’ characteristics.

characteristics	primary lung cancer (mean±SD/no.%)	brain metastasis (mean±SD/no.%)
Total(n)	70(37.6%)	116(62.4%)
gender		
Male	42 (22.6%)	43 (23.1%)
Female	28 (15.1%)	73 (39.2%)
age	51.07±10.472	57.49±9.574
AS		
low	17(9.1%)	9(4.8%)
middle	40(21.5%)	56(30.1%)
high	13(7.0%)	51(27.4%)
PAT		
squamous carcinoma	8(4.3%)	12(6.5%)
Adenocarcinoma	53(28.5%)	92(49.5%)
Neuroendocrine tumors	3(1.6%)	2(1.1%)
Lymphoepithelioma-like carcinoma	2(1.1%)	2(1.1%)
others	4(2.2%)	8(4.3%)
drunk		
yes	1(0.5%)	10(5.4%)
no	69(37.1%)	106(57.0%)
LCC	6.56±2.238	8.43±3.297
LCS		
low	39(20.1%)	23(12.4%)
middle	20(10.8%)	45(24.2%)
high	11(6.0%)	48(25.8%)
PLTC	254.59±70.729	282.64±71.682
PLTS		
low	32(17.2%)	26(14.0%)
middle	19(10.2%)	40(21.5%)
high	19(10.2%)	50(26.9%)
NRS	0.59±0.101	0.66±0.976
LRC	0.31±0.108	0.23±0.838
LRS		
low	14(7.5%)	48(25.8%)
middle	21(11.3%)	45(24.2%)
high	35(18.8%)	23(12.4%)
ERS		
low	16(8.6%)	43(23.1%)
middle	29(15.6%)	40(21.5%)
high	25(13.4%)	33(17.7%)
ALBS		
low	19(10.2%)	50(26.9%)
middle	31(16.7%)	30(16.1%)
high	20(10.8%)	36(19.4%)
GLUS		
low	32(17.2%)	37(19.9%)
middle	20(10.8%)	41(22.0%)
high	18(9.7%)	38(20.4%)
KC	3.83±0.297	3.95±0.413
KS		
low	27(14.5%)	36(19.4%)
middle	30(16.1%)	33(17.7%)
high	13(7.0%)	47(25.3%)
ClC	105.43±5.097	103.59±3.322
ClS		
low	19 (10.2%)	47(25.3%)
middle	24(13.0%)	42(22.6%)
high	27(14.5%)	27(14.5%)
FibC	3.47±1.269	4.43±1.127
FibS		
low	40(21.5%)	14(7.5%)
middle	20(10.8%)	46(24.7%)
high	10(5.4%)	56(30.1%)
TTC	16.56±0.889	16.09±1.130
TTS		
low	17(9.1%)	47(25.3%)
middle	20(10.8%)	41(22.0%)
high	33(17.7%)	28(15.1%)
T		
T1	57(30.6%)	48(25.8%)
T2	6(3.2%)	36(19.4%)
T3	5(2.7%)	21(11.3%)
T4	2(1.1%)	11(6.0%)
N		
N0	55(30.0%)	45(24.2%)
N1	15(8.1%)	71(38.2%)

### Variables selection

Univariate analysis was performed by SPSS (v26.0). The meaningful variables of single factor analysis(*p*<0.1) were introduced into logistic regression for multivariate analysis, *p*< 0.05 was statistically significant. The independent risk factors were included By R Studio (v4.2.1) to construct nomogram. C-index was used to judge the predictive capacity of nomogram. We then verified the appropriate calibration in the primary cohort and validation cohorts. The ROC curve was used to assess the nomogram. Furthermore, the DCA analysis shows that the model possesses favourable clinical practice value.

## Result

### Univariate and multivariate analysis of risk factors

Univariate analysis showed that the factors affecting brain metastasis included the following factors (**Table 2**): gender (*P*=0.001, B=-1.009), pathological type (PAT) (*P*=0.074, B=-0.253), leukocyte count (LCC) (*P*<0.001, B=0.005), Fibrinogen stage (FibS) (*P*<0.001, B=1.396).

**Table 2:** Univariate and Multivariate analysis of risk factors.

Univariate analysis of risk factors									
		B	S.E.	Wald	df	Sig.	Exp(B)	95% CI for Exp(B)	
								Lower	Upper
gender		-1.009	0.312	10.477	1	0.001	0.364	0.198	0.672
age		0.044	0.015	8.854	1	0.003	1.045	1.015	1.076
AS		0.74	0.233	10.094	1	0.001	2.096	1.328	3.308
PAT		-0.253	0.142	3.19	1	0.074	0.777	0.588	1.025
drunk		1.873	1.06	3.122	1	0.077	6.509	0.815	51.993
LCC		0.378	0.087	18.894	1	0	1.459	1.23	1.73
LCS		1.047	0.215	23.69	1	0	2.849	1.869	4.344
PLTC		0.005	0.002	6.826	1	0.009	1.005	1.001	1.009
PLTS		0.541	0.187	8.389	1	0.004	1.718	1.191	2.479
NRS		0.835	0.207	16.236	1	0	2.305	1.536	3.46
LRC		-9.191	1.93	22.678	1	0	0	0	0.004
LRS		-0.887	0.208	18.227	1	0	0.412	0.274	0.619
ERS		-0.351	0.196	3.223	1	0.073	0.704	0.48	1.033
ALBS		-0.365	0.195	3.494	1	0.062	0.694	0.473	1.018
GLUS		0.415	0.192	4.696	1	0.03	1.515	1.04	2.205
KC		0.96	0.419	5.246	1	0.022	2.611	1.148	5.936
KS		0.468	0.186	6.3	1	0.012	1.596	1.108	2.3
ClC		-0.183	0.059	9.653	1	0.002	0.833	0.742	0.935
ClS		-0.524	0.194	7.323	1	0.007	0.592	0.405	0.866
FibC		0.673	0.143	22.173	1	0	1.96	1.481	2.594
FibS		1.396	0.228	37.332	1	0	4.038	2.58	6.318
TTC		-0.354	0.148	5.745	1	0.017	0.702	0.525	0.937
TTS		-0.556	0.191	8.424	1	0.004	0.574	0.394	0.835
T		0.917	0.221	17.168	1	0	2.502	1.621	3.861
N		1.755	0.348	25.431	1	0	5.785	2.924	11.445
Multivariate analysis of risk factors									
		B	S.E.	Wald	df	Sig.	Exp(B)	95% C.I.for EXP(B)	
								Lower	Upper
Step 4^d^	gender	0.99	0.464	4.562	1	0.033	2.692	1.085	6.677
	Pathological type			15.448	5	0.009			
	Squamous cell cancer	0.361	1.017	0.126	1	0.723	1.435	0.196	10.528
	small cell carcinoma	-2.23	1.174	3.606	1	0.058	0.108	0.011	1.074
	adenosquamous carcinoma	-0.972	1.431	0.461	1	0.497	0.378	0.023	6.255
	other types	-24.185	27193.07	0	1	0.999	0	0	.
	neuroendocrine carcinoma	-1.488	1.57	0.899	1	0.343	0.226	0.01	4.897
	Leukocyte count	0.292	0.11	6.992	1	0.008	1.339	1.078	1.661
	Fib grade			30.594	2	0			
	Fib middle grade	-2.888	0.587	24.173	1	0	0.056	0.018	0.176
	Fib high grade	-0.571	0.544	1.102	1	0.294	0.565	0.194	1.641
	Constant	-0.97	1.495	0.421	1	0.516	0.379		

Introducing the significant factors of single factor analysis into Logistic regression for multivariate analysis and then we got independent risk factors as follows: gender (*P*=0.033 HR=2.692,95% CI 1.085-6.677), PAT(*P*=0.009), LCC (*P*=0.008 HR=1.339,95% CI 1.078-1.661), Fibs(*P*<0.001) (**Table 2**).

### Development of final prediction model

**Figure 1:**
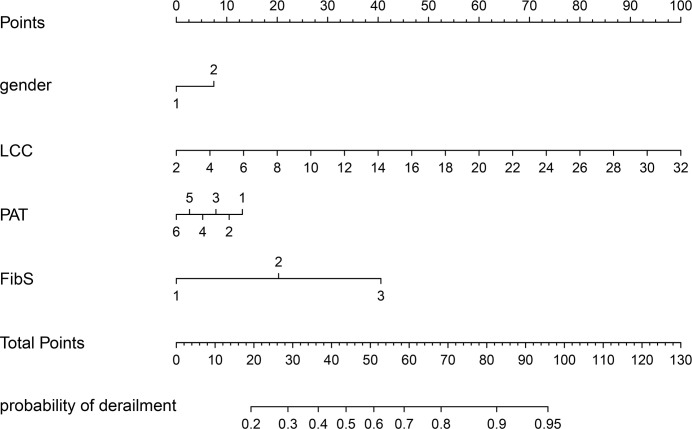
BMs-related nomogram prediction score. BMs-related nomogram was constructed to predict BMs for lung cancer patients, with the gender, PAT, LCC and FibS. The nomogram showed the probability of brain metastasis in patients with a pathological diagnosis of lung cancer. PAT, pathlogicl type; LCC, leukocyte count; FibS, Fibrinogen stage.

**Figure 2:**
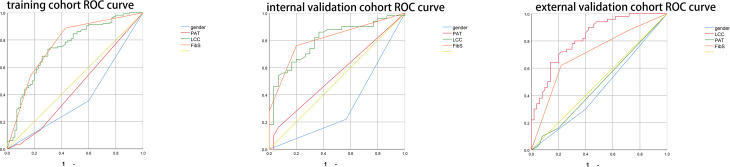
Figure 2 shows the receiver operate characteristic (ROC) curves of these risk factors for predicting the risk of brain metastasis.

**Figure 3:**
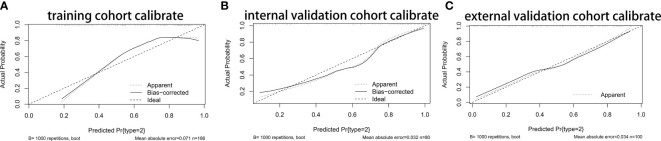
The Calibration curves of nomogram. **(A)** The Calibration curves of nomogram in primary cohort. **(B)** The Calibration curves of nomogram in internal validation cohort. **(C)** The Calibration curves of nomogram in primary cohort in external cohort.

Based on the results of multivariate analysis, we constructed a nomogram (**Figure 1**). The risk factors introduced in the model were given various weights in conformity with the degree of influence, and received different scores according to the individual information of patients. The scores are added together to obtain the ultimate scores, and the forecast results could be found in the nomogram. The C-index in this study was 0.811, showing a good prediction effect. **Figure 2** shows the receiver operating characteristic (ROC) curves of these independent risk factors for predicting the risk of brain metastasis. These factors are quite accurate in predicting brain metastasis (the area under the curve [AUCs] of those factors were 0.768, 0.746, 0.446, 0.377). Afterwards, we verified a appropriate calibration in the primary cohort and validation cohorts (**Figure 3**).

### Clinical usage

**Figure 4:**
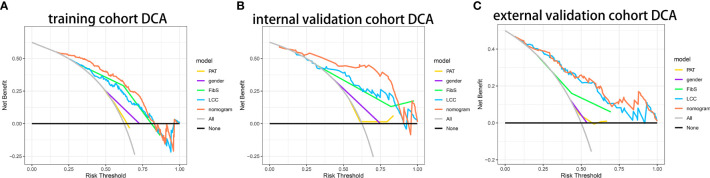
Decision curve analysis of nomogram. **(A)** The DCA curves of nomogram in primary cohort. **(B)** The DCA curves of nomogram in internal validation cohort. **(C)** The DCA curves of nomogram in primary cohort in external cohort.

**Figure 4** showed that if the threshold probability of a patient or a doctor is in the range from 0 to 0.85, the net benefit is equivalent, in accordance with DCA. The y-axis shows the net benefit, which is the different value between the ratio of false positive patients and the ratio of true positive patients, weighted by the relative harm of forgoing treatment and the negative effects of unnecessary treatment (11). The sloping glossy full line stand for the assumption that all patients have BMs. The horizontal glossy full line stands for the assumption that all patients have no BMs. The sloping dashed lines stand for all patients considered to be BMs according to the nomogram. The decision curves in cohorts shows that if the threshold probability is between 0 and 0.80, then the use of the integrated nomogram predicts that BMs will yield more benefits than treating all patients or none, while the perfect model is the model with the highest net benefit under any threshold probability.

## Discussion

In our study, the rate of brain metastasis in female is higher than that of male. To understand this situation, we must know that gender differences in human growth, development, disease, and death are so common that people almost acquiesce to gender differences as reasonable. Understanding these differences needs comprehensive analysis at the molecular, cellular, and tissue level. In recent years, this relationship has attracted more and more attention, with researchers investigating the role of gender-related molecular patterns. Thus, this adds to the understanding of fundamental gender differences, identifying mutational (12) and methylation profiles (13), followed by transcriptomes (14) and tumor metabolism (15). Analysis of cellular immanent mechanisms, central to the biology of human cancer cells, is the devitalization of RB1 and p53 functions (16). The change of p53 function may engender differential effects on males and females in multiple species. This partly explains the conclusions of our study. Preliminary characterization of mice with complete loss of p53 function suggests that they develop normally but have an increased rate of spontaneous tumor formation (17). It reflects the effect of P53 on the tumor. By using astrocytes as the cell of origin in a mouse model of glioblastoma, the loss of p53 function was found to result in significantly increased in growth *in vitro* and tumorigenesis *in vivo* of male astrocytes (18); when RB1 and p53 function are used up, both male and female astrocytes put up analogical tumor-forming potential. This evidence suggests that when P53 and RB1 are intact, gender differences in their regulation have a significant impact on malignant transformation. Both male and female astrocytes differ in cAMP synthesis and degradation, leading to differences in basal cAMP levels (19). The connection between intra-cellular cAMP and tumor growth has been established for decades (20). Analysis of non-intracellular mechanisms, first, there are tremendous differences in immunologic function between males and females, especially in autoimmune diseases. Multiple sclerosis (MS) and rheumatoid arthritis (RA) are twice as common in females compared to males (21, 22), and more than 90% of SLE cases are in females (23, 24); second, another significant and possibly cancer-related gender discrepancy is the effect of gender on vascular function. Cardiovascular disease occurs more frequently in males than females (25). The mechanistic basis for these maturing observations is dimness and may include gender discrepancy in responses to early environmental stressors and acute vascular effects of gender hormones. Possible hormone-dependent mechanisms include estrogen-irritative vasodilatory effects of nitric oxide and prostacyclin, and anti-inflammatory and antioxidant effects of estrogen (26). As mentioned earlier, P53, RB1 and cAMP have important roles in tumor growth and reproduction. The significant difference in basal cAMP between males and females contributes to the difference in the incidence of brain metastases in males and females. The specific mechanisms of P53, RB1 and cAMP affect tumor growth and reproduction need to be further investigated experimentally. In addition, the effect of gender on vascular function is evident. It is well known that the occurrence of brain metastases requires several steps: the shedding of tumor cells at the original site, entering the blood vessels, penetrating the blood-brain barrier with the blood vessels, entering the fixed value site in brain, extravasating to the brain parenchyma, and completing brain metastases. Therefore, blood vessels play a very important role in brain metastasis. As we all know, brain metastases usually occur in areas with rich blood vessels. And the difference of vascular function between male and female can explain to some extent why there are gender differences in brain metastasis.

A retrospective analysis based on the SEER database revealed that the occurrence of BM in non-small cell lung cancer was 9%, and the lowest incidence of squamous cell carcinoma in NSCLC histology was 6% (27). This conclusion is consistent with our research. Adenocarcinoma is a risk factor for brain metastasis in lung cancer. Meanwhile, another study noted that squamous cell carcinoma was significantly related to low BM occurrence in patients with non-small cell lung cancer (28).

Ekaterina Friebel et al. performed a high-parameter single-cell mapping of the tumor microenvironment of patients with brain metastasis, and the results showed that metastasis favored T cell and monocyte-derived macrophage invasion (29). Besides, inflammatory mediators are also involved in several steps of tumor metastasis. EMT,the first step of metastasis, makes migration possible (30). EMT can be enhanced by activating the NF-kB/STAT3 pathway and producing inflammatory cytokines (31). The extracellular matrix (ECM) is then modified, particularly through inflammatory mediators, to allow cancer cells to enter blood vessels and lymphatic vessels (32). Finally, extravasation of cancer cells is induced by chemokines (a family of pro-inflammatory mediators) (33). Then, the process of BMs is completed. Neutrophils accounts for 50-70% of all leukocytes (34) and can mirror the condition of host inflammation, a hallmark of cancer (16). The influence of neutrophils in cancer is multifactorial and still not know it inside out. They can participate in different periods of the carcinogenic process, including tumor origination, growth, propagation, or metastatic spread (35, 36). The release of reactive oxygen species (ROS), reactive nitrogen species (RNS) or proteases from neutrophils can promote tumorigenesis and metastasis (37). Neutrophils can promote tumor propagation and metastasis by destroying the immune system. Moreover, insulin receptor substrate 1 (IRS1) activation of PI3K signaling also mediates tumor propagation as neutrophil elastase metastasizes to cancer cells (38). Ultimately, neutrophils can also promote metastatic spread by repressing natural killer function and promoting exosmosis of tumor cells (39, 40). As seen here, the role of neutrophils in cancer spread is complicated.

Fibrinogen, the most ample plasma coagulation factor, is synthesized by hepatocytes. Animal experiments have proved that increasing local coagulation function will lead to an increase in BMs. This is the same with our conclusion (41). Fibrinogen has been revealed to have prognostic implications in a variety of cancers, including lung cancer (42–45). There is increasing evidence of a correlation between fibrinogen and metastatic spread (46, 47). The process of BMs is divided into: tumor cells oozing from the primary site, tumor cells entering the vasculature, tumors reaching the brain with the vasculature and tumor cells oozing from the vasculature to the brain parenchyma to complete the BMs. Elevated serum fibrinogen concentrations alter blood viscosity, rheology, and endothelial function, which may increase the probability of tumor cells exuding from the vasculature to the brain parenchyma and thus promote BMs. Fibrinogen can enhance brain tumor-initiating cells (BTICs) intercellular adhesion and improve the motility of BTIC, thereby increasing the invasiveness of BTIC (48). Clinical studies have shown that prophylactic anticoagulation in lung cancer patients does not increase the risk of bleeding (49–51). Can lung cancer patients use anticoagulation prophylactically to reduce the BMs? More basic and clinical studies are needed to confirm our conjecture.

Even though our nomogram displays encouraging results among the cohorts, there are still certain shortcomings. Due to the retrospective method is adopted in this study, inherent deviations such as selection deviation and detection deviation is inevitably occur. Furthermore, continuous monitoring of changes in certain parameters cannot be completed. In addition, molecular mechanism researches and large-scale and multi-center clinical trials are required to revise the model.

## Conclusion

This research provided a better understanding of the risk factors for brain metastases among lung cancer patients. Besides, we have developed a new pragmatic nomogram, which immensely extends the range of clinical practice to calculate the characteristics of patients with brain metastases from lung cancer.

## Data availability statement

The original contributions presented in the study are included in the article/supplementary material. Further inquiries can be directed to the corresponding authors.

## Ethics statement

This study was approved by the Guangdong Provincial People’s Hospital. Written informed consent from the patients was not required to participate in this study in accordance with the national legislation and the institutional requirements.

## Author contributions

All authors listed have made a substantial, direct, and intellectual contribution to the work and approved it for publication.

## References

[1] Schouten LJ Rutten J Huveneers HA Twijnstra A. Incidence of brain metastasis in a cohort of patients with carcinoma of the breast, colon, kidney, and lung and melanoma. Cancer (2002) 94:2698–705. doi: 10.1002/cncr.10541 12173339

[2] Saad AG Yeap BY Thunnissen FB Pinkus GS Pinkus JL Loda M. Immunohistochemical markers associated with brain metastasis in patients with nonsmall cell lung carcinoma. Cancer (2008) 113:2129–38. doi: 10.1002/cncr.23826 PMC2597625 18720359

[3] Smith DR Bian Y Wu CC Saraf A Tai CH Nanda T. Natural history, clinical course and predictors of interval time from initial diagnosis to developmalet of subsequent NSCLC brain metastasis. J Neurooncol (2019) 143:145–55. doi: 10.1007/s11060-019-03149-4 30874953

[4] Waqar SN Samson PP Robinson CG Bradley J Devarakonda S Du L. Non-small-cell lung cancer with brain metastasis at presentation. Clin Lung Cancer (2018) 19:e373–9. doi: 10.1016/j.cllc.2018.01.007 PMC6990432 29526531

[5] Bajard A Westeel V Dubiez A. Multivariate analysis of factors predictive of brain metastasis in localised non-small cell lung carcinoma. Lung Cancer (2004) 45:317–23. doi: 10.1016/j.lungcan.2004.01.025 15301872

[6] Ceresoli GL Reni M Chiesa G Carretta A Schipani S Passoni P. Brain metastasis in locally advanced nonsmall cell lung carcinoma after multimodality treatmalet:risk factors analysis. Cancer (2002) 95:605–12. doi: 10.1002/cncr.10687 12209754

[7] Robnett TJ Machtay M Stevenson JP Algazy KM Hahn SM. Factors affecting the risk of brain metastasis after definitive chemoradiation for locally advanced non-small-cell lung carcinoma. J Clin Oncol (2001) 19:1344–9. doi: 10.1200/JCO.2001.19.5.1344 11230477

[8] Chen AM Jahan TM Jablons DM Garcia J Larson DA. Risk of cerebral metastasis and neurological death after pathological complete response to neoadjuvant therapy for locally advanced non-small-cell lung cancer: clinical implications for the subsequent managemalet of the brain. Cancer (2007) 109:1668–75. doi: 10.1002/cncr.22565 17342770

[9] Davis FG Dolecek TA McCarthy BJ Villano JL. Toward determining the lifetime occurrence of metastatic brain tumors estimated from 2007 united states cancer incidence data. Neuro-Oncology (2012) 14(9):1171–7. doi: 10.1093/neuonc/nos152 PMC3424213 22898372

[10] Lee DS Kim YS Jung SL Lee KY Kang JH Park S. The relevance of serum carcinoembryonic antigen as an indicator of brain metastasis detection in advanced non-small cell lung cancer. Tumour Biol (2012) 33(4):1065–73. doi: 10.1007/s13277-012-0344-0 22351560

[11] Zhang GH Liu YJ De Ji M. Risk factors, prognosis, and a new nomogram for predicting cancer-specific survival among lung cancer patients with brain metastasis: a retrospective study based on SEER. Lung (2022) 200(1):83–93. doi: 10.1007/s00408-021-00503-0 35067758

[12] Vickers AJ Cronin AM Elkin EB Gonen M. Extensions to decision curve analysis, a novel method for evaluating diagnostic tests, prediction models and molecular markers. BMC Med Inform Decis Mak (2008) 8:53. doi: 10.1186/1472-6947-8-53 19036144 PMC2611975

[13] Zhang H Liao J Zhang X Zhao E Liang X Luo S. Gender difference of mutation clonality in diffuse glioma evolution. Neuro Oncol (2019) 21:201–13. doi: 10.1093/neuonc/noy154 PMC6374767 30256978

[14] Yang W Warrington NM Taylor SJ Whitmire P Carrasco E Singleton KW. Gender differences in GBM revealed by analysis of patient imaging, transcriptome, and survival data. Sci T ransl Med (2019) 11(473):eaao5253. doi: 10.1126/scitranslmed.aao5253 PMC6502224 30602536

[15] Ippolito JE Yim AK Luo J Chinnaiyan P Rubin JB. Genderual dimorphism in glioma glycolysis underlies gender differences in survival. JCI Insight (2017) 2. doi: 10.1172/jci.insight.92142 PMC5543918 28768910

[16] Hanahan D Weinberg RA. Hallmarks of cancer: the next generation. Cell (2011) 144(5):646–74. doi: 10.1016/j.cell.2011.02.013 21376230

[17] Donehower LA Harvey M Slagle BL McArthur MJ Montgomery CA Jr Butel JS. Mice deficient for p53 are developmaletally normal but susceptible to spontaneous tumours. Nature (1992) 356(6366):215–21. doi: 10.1038/356215a0 1552940

[18] Sun T Warrington NM Luo J Brooks MD Dahiya S Snyder SC. Genderually dimorphic RB inactivation underlies mesenchymal glioblastoma prevalence in males. J Clin Invest (2014) 124(9):4123–33. doi: 10.1172/JCI71048 PMC4151215 25083989

[19] Warrington NM Sun T Luo J McKinstry RC Parkin PC Ganzhorn S. The cyclic AMP pathway is a gender-specific modifier of glioma risk in type I neurofibromatosis patients. Cancer Res (2015) 75(1):16–21. doi: 10.1158/0008-5472.CAN-14-1891 25381154 PMC4286430

[20] Racagni G Pezzotta S Giordana MT Iuliano E Mocchetti I Spanu G. Cyclic nucleotides in experimaletal and human brain tumors. J Neurooncol (1983) 1(1):61–7. doi: 10.1007/BF00153643 6086852

[21] Sellner J Kraus J Awad A Milo R Hemmer B Stüve O. The increasing incidence and prevalence of female multiple sclerosis–a critical analysis of potential environmaletal factors. Autoimmun Rev (2011) 10(8):495–502. doi: 10.1016/j.autrev.2011.02.006 21354338

[22] van Vollenhoven RF. Gender differences in rheumatoid arthritis: more than meets the eye. BMC Med (2009) 7:12. doi: 10.1186/1741-7015-7-12 19331649 PMC2670321

[23] Hamilton A Sibson NR. Role of the systemic immune system in brain metastasis. Mol Cell Neurosci (2013) 53:42–51. doi: 10.1016/j.mcn.2012.10.004 23073146

[24] Weckerle CE Niewold TB. The unexplained female predominance of systemic lupus erythematosus: clues from genetic and cytokine studies. Clin Rev Allergy Immunol (2011) 40(1):42–9. doi: 10.1007/s12016-009-8192-4 PMC2891868 20063186

[25] Lehto HR Lehto S Havulinna AS Salomaa V. Does the clinical spectrum of incident cardiovascular disease differ between male and female? Eur J Prev Cardiol (2014) 21(8):964–71. doi: 10.1177/2047487313482284 23482728

[26] Resanovic I Rizzo M Zafirovic S Bjelogrlic P Perovic M Savic K. Anti-atherogenic effects of 17β-estradiol. Horm Metab Res (2013) 45(10):701–8. doi: 10.1055/s-0033-1343478 23681753

[27] Goncalves PH Peterson SL Vigneau FD Shore RD Quarshie WO Islam K. Risk of brain metastasis in patients with nonmetastatic lung cancer: analysis of the metropolitan Detroit surveillance, epidemiology, and end results (SEER) data. Cancer (2016) 122(12):1921–7. doi: 10.1002/cncr.30000 PMC4892979 27062154

[28] Sun DS Hu LK Cai Y Li XM Ye L Hou HY. A systematic review of risk factors for brain metastasis and value of prophylactic cranial irradiation in non-small cell lung cancer. Asian Pac J Cancer Prev (2014) 15(3):1233–9. doi: 10.7314/APJCP.2014.15.3.1233 24606446

[29] Friebel E Kapolou K Unger S Núñez NG Utz S Rushing EJ. Single-cell mapping of human brain cancer reveals tumor-specific instruction of tissue-invading leukocytes. Cell (2020) 181(7):1626–42.e20. doi: 10.1016/j.cell.2020.04.055 32470397

[30] Kalluri R Weinberg RA. The basics of epithelial mesenchymal transition. J Clin Invest (2009) 119:1420e8. doi: 10.1172/JCI39104 19487818 PMC2689101

[31] Grivennikov SI Karin M. Inflammation and oncogenesis: a vicious connection. Curr Opin Genet Dev (2010) 20:65e71. doi: 10.1016/j.gde.2009.11.004 20036794 PMC2821983

[32] Nguyen DX Bos PD Massague J. Metastasis: from dissemination to organ specific colonization. Nat Rev Cancer (2009) 9:274e84. doi: 10.1038/nrc2622 19308067

[33] Mantovani A Allavena P Sica A Balkwill F. Cancer-related inflammation. Nature (2008) 454:436e44. doi: 10.1038/nature07205 18650914

[34] Bronte V Brandau S Chen SH Colombo MP Frey AB Greten TF. Recommaledations for myeloid-derived suppressor cell nomaleclature and characterization standards. Nat Commun (2016) 7:12150. doi: 10.1038/ncomms12150 27381735 PMC4935811

[35] Swierczak A Mouchemore KA Hamilton JA Anderson RL. Neutrophils: important contributors to tumor progression and metastasis. Cancer Metastasis Rev (2015) 34(4):735–51. doi: 10.1007/s10555-015-9594-9 26361774

[36] Coffelt SB Wellenstein MD de Visser KE. Neutrophils in cancer: neutral no more. Nat Rev Cancer (2016) 16(7):431–46. doi: 10.1038/nrc.2016.52 27282249

[37] Antonio N Bønnelykke-Behrndtz ML Ward LC Collin J Christensen IJ Steiniche T. The wound inflammatory response exacerbates growth of pre-neoplastic cells and progression to cancer. EMBO J (2015) 34(17):2219–36. doi: 10.15252/embj.201490147 PMC4585460 26136213

[38] Houghton AM Rzymkiewicz DM Ji H Gregory AD Egea EE Metz HE. Neutrophil elastase-mediated degradation of IRS-1 accelerates lung tumor growth. Nat Med (2010) 16(2):219–23. doi: 10.1038/nm.2084 PMC2821801 20081861

[39] Welch DR Schissel DJ Howrey RP Aeed PA. Tumor-elicited polymorphonuclear cells, in contrast to "normal" circulating polymorphonuclear cells, stimulate invasive and metastatic potentials of rat mammary adenocarcinoma cells. Proc Natl Acad Sci U S A (1989) 86(15):5859–63. doi: 10.1073/pnas.86.15.5859 PMC297730 2762301

[40] Spiegel A Brooks MW Houshyar S Reinhardt F Ardolino M Fessler E. Neutrophils suppress intraluminal NK cell-mediated tumor cell clearance and enhance extravasation of disseminated carcinoma cells. Cancer Discovery (2016) 6(6):630–49. doi: 10.1158/2159-8290.CD-15-1157 PMC4918202 27072748

[41] Feinauer MJ Schneider SW Berghoff AS Robador JR Tehranian C Karreman MA. Local blood coagulation drives cancer cell arrest and brain metastasis in a mouse model. Blood (2021) 137(9):1219–32. doi: 10.1182/blood.2020005710 33270819

[42] Buccheri G Ferrigno D Ginardi C Zuliani C. Haemostatic abnormalities in lung cancer: prognostic implications. Eur J Cancer (1997) 33(1):50–5. doi: 10.1016/S0959-8049(96)00310-3 9071899

[43] Pavey SJ Hawson GA Marsh NA. Impact of the fibrinolytic enzyme system on prognosis and survival associated with non-small cell lung carcinoma. Blood Coagul Fibrinolysis (2001) 12:51–8. doi: 10.1097/00001721-200101000-00008 11229827

[44] Seitz R Rappe N Kraus M Immel A Wolf M Maasberg M. Activation of coagulation and fibrinolysis in patients with lung cancer: relation to tumour stage and prognosis. Blood Coagul Fibrinolysis (1993) 4:249–54. doi: 10.1097/00001721-199304000-00006 8388741

[45] Wojtukiewicz MZ Zacharski LR Moritz TE Hur K Edwards RL Rickles FR. Prognostic significance of blood coagulation tests in carcinoma of the lung and colon. Blood Coagul Fibrinolysis (1992) 3:429–37. doi: 10.1097/00001721-199203040-00010 1330024

[46] Jones JM McGonigle NC McAnespie M Cran GW Graham AN. Plasma fibrinogen and serum c-reactive protein are associated with non-small cell lung cancer. Lung Cancer (2006) 53:97–101. doi: 10.1016/j.lungcan.2006.03.012 16698114

[47] Zhao J Zhao M Jin B Yu P Hu X Teng Y. Tumor response and survival in patients with advanced non-small-cell lung cancer: the predictive value of chemotherapy-induced changes in fibrinogen. BMC Cancer (2012) 12:330. doi: 10.1186/1471-2407-12-330 22852778 PMC3492194

[48] Dzikowski L Mirzaei R Sarkar S Kumar M Bose P Bellail A. Fibrinogen in the glioblastoma microenvironmalet contributes to the invasiveness of brain tumor-initiating cells. Brain Pathol (2021) 31(5):e12947. doi: 10.1111/bpa.12947 33694259 PMC8412081

[49] Ek L Gezelius E Bergman B Bendahl PO Anderson H Sundberg J. Swedish Lung cancer study group (SLUSG). randomized phase III trial of low-molecular-weight heparin enoxaparin in addition to standard treatmalet in small-cell lung cancer: the RASTEN trial. Ann Oncol (2018) 29(2):398–404. doi: 10.1093/annonc/mdx716 29106448 PMC5834130

[50] Wood P Boyer G Mehanna E Cagney D Lamba N Catalano P. Intracerebral haemorrhage in patients with brain metastasis receiving therapeutic anticoagulation. J Neurol Neurosurg Psychiatry (2021) 9:jnnp–2020-324488. doi: 10.1136/jnnp-2020-324488 33687972

[51] Leader A Hamulyák EN Carney BJ Avrahami M Knip JJ Rozenblatt S. Intracranial hemorrhage with direct oral anticoagulants in patients with brain metastasis. Blood Adv (2020) 4(24):6291–7. doi: 10.1182/bloodadvances.2020003238 PMC7756985 33351124

